# Treatment options in obstructive sleep apnea

**DOI:** 10.1007/s11739-022-02983-1

**Published:** 2022-04-23

**Authors:** Francesco Gambino, Marta Maria Zammuto, Alessandro Virzì, Giosafat Conti, Maria Rosaria Bonsignore

**Affiliations:** 1 Division of Pulmonary Medicine, Ospedali Riuniti Villa Sofia-Cervello, Via Trabucco 180, 90146 Palermo, Italy; 2 PROMISE Department, University of Palermo, Palermo, Italy

**Keywords:** Personalized medicine, Clinical phenotypes, Physiological phenotypes, CPAP adherence, Non-PAP treatment

## Abstract

Treatment of OSA with CPAP is currently the recommended treatment and has the greatest evidence of efficacy on AHI, symptoms and comorbidities. Symptomatic patients with moderate-severe OSA generally have good adherence to CPAP therapy, while those with mild OSA, female, young and generally paucisymptomatic, have lower CPAP adherence, especially in the medium and long term. The recent identification of different clinical and pathophysiological phenotypes of OSA has paved the way for alternative treatments to CPAP, leading to an increasingly personalized therapy. Weight loss and lifestyle modifications are highly recommended in all obese or overweight patients. Mandibular advancement devices (MAD), positional therapy (PT) and hypoglossal nerve stimulation (HSN) are recent and personalized alternative therapies on which there is promising and encouraging data but with still little strong scientific evidence. The purpose of this review is to compare the efficacy, adherence and costs of various therapeutic options for OSA patients in the light of recent evidence and to provide useful guidance for specialists.

## Introduction

Obstructive sleep apnea (OSA) is a highly prevalent disease characterized by the occurrence of partial or complete upper airway collapse during sleep [1]. Obstructive respiratory events acutely result in cyclic intermittent hypoxia and sleep fragmentation. If left untreated, OSA negatively impacts daytime function and increases the risk of motor vehicle accidents. The most common symptoms are intermittent snoring, disturbed sleep, daytime sleepiness, and fatigue. OSA contributes to systemic hypertension through sympathetic hyperactivation and may be associated with cognitive impairment [1]. OSA patients are often obese, show multiple cardiometabolic comorbidities, and a profile of high cardiovascular risk [1, 2]. The most common treatment of OSA is continuous positive airway pressure (CPAP) applied every night during sleep through a nasal mask to splint the upper airway open [3]. However, over time, the interest in alternative treatments has increased, especially because acceptance of CPAP by the patients is often low. Recent research has clarified that many clinical and pathophysiological phenotypes of OSA exist, opening the way to personalized treatment of the disease [4]. Among clinical phenotypes, the simplest classification includes three clusters of OSA presentation: patients with excessive daytime sleepiness (EDS), patients with disturbed sleep, and minimally symptomatic patients [5]. As for pathophysiological phenotypes, besides anatomic impairment of the upper airway, additional functional traits have been identified, leading to a rational therapeutic approach, including non-CPAP treatment(s) [4]. While a personalized therapeutic approach is a major goal of current research, additional aspects of OSA treatment should be taken into consideration. Each physician ultimately suggests a treatment based not only on the evidence of its effectiveness but also on the disease phenotype, the availability, the cost and the acceptance by the patient. OSA treatment is no exception, and this review will try to provide the reader with a concise overview of the most common treatment options and their clinical indications in adult OSA patients. Because of space limitations, upper airway surgery will not be considered since a recent American Academy of Sleep Medicine guideline did not recommend upper airway surgery as a first-line treatment for severe OSA, although the surgical option(s) should be discussed with the patient [6, 7].

## CPAP

CPAP is the mainstay of OSA treatment, especially in patients with moderate-severe disease based on the frequency of respiratory events during sleep, i.e., the apnea–hypopnea index (AHI). CPAP is indicated for AHI > 15/h independent of symptoms, or for lower AHI associated with EDS [8]. Its effectiveness is evidence-based, since CPAP treatment in adults with OSA significantly decreases disease severity, sleepiness, blood pressure, and the risk for motor vehicle accidents, and improves sleep-related quality of life [9]. The therapeutic pressure has to be established for each patient based on the results of manual titration, or more often home titration with an automatic CPAP device over a few nights. The mask should fit well without leaks, and nasal masks should be preferred to oronasal masks [10].

There is a current debate on the use of automatic CPAP versus fixed pressure CPAP devices. Air leaks were unaffected by the type of CPAP [11], but in some studies fixed CPAP was superior to automatic CPAP in cardiovascular risk reduction [12] and preservation of renal function [13] during follow-up. A randomized trial is ongoing and will assess sympathetic nerve activation in response to both treatment types [14].

Compliance with CPAP is a well-known problem, especially over the medium- and long-term. The minimum Medicare criteria for CPAP reimbursement is nightly use of at least 4 h for 70% of the nights, but ideally patients should use CPAP for the entire sleep period. CPAP effectiveness in decreasing sleepiness shows a dose–response relationship [15]. Availability of long-term compliance data, found also on digital platforms by CPAP manufacturers, identified different patterns of CPAP use, with about 50% of patients being good users [16]. Good adherence to CPAP is usually found in OSA patients with EDS and significant limitations of daytime function, whereas patients referring insomnia [17] or females, mildly symptomatic patients, and patients with comorbidities showed a low likelihood of CPAP treatment success [18]. A health/safety risk behavior has also been associated with CPAP discontinuation [19], highlighting the complexity of factors in different domains which play a role in CPAP acceptance by the patients.

Some patients with indication for CPAP  treatment and poor tolerance to CPAP may gain benefit from shifting to bilevel ventilation during sleep, as recently found in a large cohort of “real-life” patients followed by using a telemedicine platform [20]. Similarly, the use of automatic bilevel ventilation may improve the clinical outcome in patients with poor tolerance to CPAP or overlap OSA-COPD syndrome [21]. Telemedicine is useful and cost-effective for the management of OSA patients on CPAP [22] and its use has greatly increased during the pandemic, likely setting a new standard of care for the future [23].

## Lifestyle changes and weight loss

OSA is highly prevalent in overweight or obese subjects, and obesity is a major risk factor for OSA [1]. Accordingly, weight loss is associated with a decrease in AHI and OSA severity [24]. A longitudinal study in the Wisconsin Sleep Cohort reported that weight gain by 10% predicted a 32% increase in AHI; conversely, a 10% weight loss was associated with a 26% decrease in AHI [25]. Therefore, weight loss should always be advised for overweight/obese patients with OSA, although it rarely causes the complete disappearance of OSA [26]. In addition, weight loss may also be effective for patients with mild OSA. Moreover, a randomized controlled trial comparing the effects of CPAP, weight loss, and weight loss + CPAP on insulin resistance, blood pressure, and C-reactive protein concentration as markers of inflammation reported larger beneficial changes when CPAP was associated with a hypocaloric diet [27]. The American Thoracic Society guideline recommended that OSA patients should participate in a comprehensive lifestyle intervention program, including exercise [27]. For severely obese OSA patients who do not succeed to lose weight, referral for bariatric surgery evaluation is suggested in the absence of contraindications [6, 27]. Studies on medications such as liraglutide and gliflozins have been conducted in diabetic OSA patients and may positively modify the cardiometabolic risk profile besides their effect on weight loss [28, 29].

## Positional therapy

Some OSA patients show more frequent and severe respiratory events when lying supine, while very few events occur while sleeping in other positions. Positional OSA (POSA) is defined as a ratio of respiratory events in the supine to non-supine position greater than 2:1, and some patients show OSA exclusively when supine (e-POSA). In a large retrospective study, the prevalence of POSA was over 50%, and of e-POSA was 20% [30]. These patients often show suboptimal compliance to CPAP treatment [30] and may be treated with devices preventing the supine position during sleep by causing vibrations in the neck or in the chest. Meta-analyses confirmed their effectiveness in preventing the supine position and reducing the AHI, although the effect was lower than CPAP [31, 32]. Short-term compliance was satisfactory, while regular use at 6 months was reported in 41.6% of the patients, with a larger therapeutic effect in patients with mild-moderate OSA than in patients with severe OSA [33].

## Mandibular advancement device (MAD)

The principle of MAD is that advancement of the mandible enlarges and stabilizes the upper airway, decreasing snoring and the occurrence of obstructive respiratory events [34, 35]. Custom-made, dual-block MAD represents an established, effective and attractive option in primary snoring, and in patients with OSA not accepting CPAP treatment [36]. Custom, titratable devices should be preferred to non-custom ones, and patients should undergo regular follow-up by both the sleep physician and the dentist [36].

According to several meta-analyses, CPAP is more effective compared to MAD in decreasing AHI and ODI [37] or daytime sleepiness [38]. However, because compliance to treatment is higher for MAD than for CPAP, MAD is considered a good alternative to CPAP treatment. Some phenotypic features of OSA have been identified as predictors of a positive response to MAD: young age, female gender, absence of severe obesity, a small neck circumference, and an AHI in the mild-moderate range; from the anatomic point of view, retraction of maxilla and mandible, a narrow airway and a short soft palate also predicted success [39]. A recent study using drug-induced sleep endoscopy (DISE) showed that tongue base collapse represents the only phenotype predicting a good response to MAD [40]. According to another study, mild to moderate upper airway collapsibility and favorable functional traits may predict a good response to MAD [41]. A high degree of upper airway collapsibility, estimated as a CPAP therapeutic pressure > 10.5 cmH_2_O, was also found to predict poor response to MAD [42]. More recently, a high ventilatory instability during sleep, i.e. a high loop gain, was reported in patients who are poorly responsive to MAD [43].

Some studies have tested the efficacy of MAD in severe OSA, which is not traditionally considered as a good indication of MAD. An observational study on patients with severe OSA refusing CPAP treatment reported positive results with MAD [44], similarly a multicenter Korean study reported a decrease in AHI by 64 ± 26% after one month of treatment, but the positive effects were especially seen in patients with low BMI [45]. Finally, an individual patient meta-analysis compared the outcomes of CPAP and MAD in patients with severe OSA from 4 randomized controlled trials and found that titratable MAD was less effective than CPAP on AHI, but results were similar for improvement in quality of life, sleepiness, and sleep macrostructure [46].

Side effects of MAD are usually minor and self-limiting at the start of treatment but can involve bite changes in long-term treatment [35, 47]. Additionally, OSA may worsen over time despite MAD use, suggesting that it may be beneficial to perform periodic control visits, especially after long-term use.

Little evidence is available as the effects of MAD on markers of cardiovascular risk are concerned. Randomized controlled trials reported no effect of MAD treatment on blood pressure, endothelial cell function, or inflammatory markers [48–51]. However, some studies documented positive cardiovascular effects after MAD treatment, such as reversal of left ventricular remodeling [52], and changes in heart rate variability [53].

## Hypoglossal nerve stimulation (HSN)

HSN was introduced in 2014 for the treatment of OSA. The hypoglossal nerve contains only motor fibers innervating several muscles including the genioglossus (GG), the main pharyngeal dilator muscle. There are monolateral devices approved for clinical use, and one device producing bilateral stimulation of the GG; the reader is referred to an extensive review for technical details [54]. HSN is indicated in adult patients intolerant of CPAP, with moderate-severe OSA, i.e. AHI ≥ 15/h and < 65/h, no more than 25% of central or mixed events, BMI < 35 kg/m^2^, and absence of complete concentric palatal collapse at DISE [55, 56]. Implantation of the device requires surgical intervention by an ENT specialist. Results have been promising and stable over time, and patients’ adherence and satisfaction are high [57]. However, efforts are being made to identify predictors of a positive response, and HSN is far from being completely clarified as pathophysiological effects and outcomes are concerned [58]. A recent study identified the physiological traits predictive of a good response to treatment: a low arousal threshold and loop gain, and occurrence of muscle compensation [59]. The HSN treatment has revived interest in transcutaneous electrical stimulation, which would have the advantage of eliminating surgery, but is less effective than HSN [60, 61].

## How to choose the right treatment for each patient?

Choosing the right treatment for each patient is a major challenge because clusters based on clinical characteristics or endotypic traits are known to exist, and physicians should suggest the best therapeutic option and personalize treatment [62]. Besides the treatments for OSA considered in this review, there are additional therapeutic options currently under study, including drugs and myofunctional therapy [62]. In addition, the possibility to combine treatment options to improve clinical and pathophysiological outcomes is very attractive but requires careful studies that put together what is known on the response to treatment of clinical phenotypes and endotypes of OSA, possibly using artificial intelligence approaches [62]. Indeed, the recently published ERS updated guideline on non-PAP therapies reported low or very low certainty of the evidence for all treatments examined [63]. Finally, although DISE is a precious tool to study upper airway functional abnormalities [64, 65], its clinical use is limited to some, not all, sleep Centers.

Table 1 summarizes the different factors to take into consideration when discussing OSA treatments alternative to CPAP with the patient. The cost of treatments is variable since Health systems apply different reimbursement rules in Europe, and this is an additional factor to consider, potentially generating health inequities [63]. It is likely that clinical research will proceed quickly towards the development of new and carefully tested clinical algorithms to help the physician and the patient to make the best choice. Patient preference is also a major variable to consider.

**Table 1:** Summary of factors to take into account in choosing among OSA treatments

Treatment	Indication	Effectiveness	Compliance	Patient preference	Cost
CPAP	Moderate-severe OSA	High	High in patients severely symptomatic for EDS, low in females and patients with mild or no symptoms	Low	Low
Weight loss-lifestyle changes	All overweight/obese OSA patients; bariatric surgery in severely obese patients	Weight loss and lifestyle changes associated with a decrease in OSA severity; bariatric surgery highly effective	Weight loss and lifestyle changes are hard to maintain in the long-term	–	Low for weight loss and lifestyle changes; high for bariatric surgery
Positional therapy	Patients with respiratory events occurring mostly or exclusively in the supine position	High especially in mild cases	High, but little data in the long-term	High	Moderate
Mandibular advancement device (MAD)	Patients with mild-moderate OSA; tongue-base collapse at DISE; refusal of CPAP	Lower than CPAP, but compensated by a longer nightly use	High	High	Moderate
Hypoglossal nerve stimulation	Patients with moderate-severe OSA refusing CPAP, not severely obese, and with < 25% of central-mixed events	High in responders (between 40 and 50% of the patients studied)	High	High	High

## Conclusions

In patients with mild OSA and in patients with predominant functional as opposed to anatomical impairment, alternative OSA treatment should be considered, especially because acceptance of CPAP in these patients is usually low. In patients with moderate-severe OSA refusing CPAP treatment, alternative treatments ought to be taken into consideration according to the clinical and physiological phenotypes and patient preferences. Ongoing work will further clarify how to personalize OSA treatment, but currently, the evidence for non-PAP therapies is insufficient to draw conclusions. On the other hand, the number of patients requesting non-CPAP treatment is high, and patient preferences can affect the choice. Results of treatment should be objectively documented, and follow-up should be regular for all types of treatment, CPAP or non-CPAP, to adjust treatment if needed.
